# Maternal age and the risk of adverse pregnancy outcomes: a retrospective cohort study

**DOI:** 10.1186/s12884-019-2400-x

**Published:** 2019-07-23

**Authors:** Ambrogio P. Londero, Emma Rossetti, Carla Pittini, Angelo Cagnacci, Lorenza Driul

**Affiliations:** 1 Clinic of Obstetrics and Gynecology, DAME, University of Udine, Academic Hospital of Udine, Piazza Santa Maria della Misericordia, 15, 33100 Udine, Italy; 2 Unit of Neonatology, Academic Hospital of Udine, 33100 Udine, Italy

**Keywords:** Pregnancy-related hypertensive disorders, Fetal periventricular leucomalacia, Fetal intraventicular hemorrhage, Maternal age

## Abstract

**Background:**

The increased potential for negative pregnancy outcomes in both extremes of reproductive age is a well-debated argument. The aim of this study was to analyze the prevalence and the outcome of pregnancies conceived at extreme maternal ages.

**Methods:**

This retrospective study considered all single consecutive pregnancies delivered in a tertiary referral center between 2001 and 2014. Patients were categorized into 4 groups according to maternal age at delivery (< 17 years; 18–28 years; 29–39 years; > 40 years). The following outcomes were considered (amongst others): pregnancy-related hypertensive disorders (PRHDs), neonatal resuscitation (NR), neonatal intensive care unit (NICU) admission, periventricular leucomalacia (PVL), and grade 3 and 4 intraventicular hemorrhage (IVH).

**Results:**

During the considered period 22,933 single pregnancies gave birth in our unit. We observed 71 women aged < 17 years, and 1552 aged > 40 years. In each year throughout the study period, there was a significant increment in maternal age of 0.041 years (95% CI 0.024–0.058) every new year. Multivariate analysis concluded out that maternal age over 40 years was an independent risk factor for preterm delivery (OR 1.36 95% CI 1.16–1.61, *p* < 0.05, PRHDs (OR 2.36 95% CI 1.86–3.00, *p* < 0.05), GDM (OR 1.71 95% CI 1.37–2.12, *p* < 0.05) cesarean section (OR 1.99 95% CI 1.78–2.23, *p* < 0.05), abnormal fetal presentation (OR 1.29 95% CI 1.03–1.61, *p* < 0.05), and fetal PVL (OR 3.32 95% CI 1.17–9.44, *p* < 0.05). We also observed that maternal age under 17 years or over 40 years was an independent risk factor for grade 3 or 4 neonatal IVH (OR 2.97 95% CI 1.24–7.14, *p* < 0.05).

**Conclusions:**

These findings confirm a negative impact of extreme maternal ages on pregnancy. These results should be carefully taken into account by maternal care providers in order to inform women adequately, supporting them in understanding potential risks associated with their procreation choices, and to improve clinical surveillance.

**Electronic supplementary material:**

The online version of this article (10.1186/s12884-019-2400-x) contains supplementary material, which is available to authorized users.

## Background

Maternal age at childbearing has dramatically shifted in the last decades due to a broad range of social and cultural determinants. In Italy the mean age at delivery rose from 25.2 years in 1981 to 31.7 in 2015 [1, 2]. This trend toward delayed childbearing is reported worldwide (e.g. USA or China) [3–5], and comes, in parallel, with a decline in pregnancies at a younger age, so that these are increasingly rare in developing countries. The teen birth rate in the USA has fallen 61% from 1991 [5].

Both extremes of the reproductive age are considered at risk for adverse pregnancy outcomes. Teenage mothers have a higher risk of preterm birth, low birth weight, low Apgar score and postnatal mortality [6]. Whether this association is determined by a biological immaturity or rather by socio-economic disadvantages, behavioural factors or lack of access to high quality antenatal care is still a topic for much discussion. [7–9]. On the other hand delayed childbearing carries a higher risk of maternal and obstetric complications. The concern for the “elderly primigravida” was first published in 1950 [10]; since then many researchers investigated the effect of aging on birth outcome. The majority of studies report an association between advanced maternal age and preterm delivery, low birth weight, perinatal death, and cesarean section [11–16]. There are some studies, however, that fail to demonstrate such unfavourable conclusions [4, 17–19]. And an emerging third category even demonstrates positive outcomes, with an example being a recent retrospective study from China which found a lower risk of adverse fetal outcome for older mothers [4]. The impact of presumed social and economic advantages of older women outweighing biological vulnerability, advocated by some, still needs to be conclusively proven. It is however worth noting, that although infants born to mothers over 40 years of age are more frequently admitted to intensive neonatal care, these pregnancies tend to be associated with improved perinatal care outcomes over time [20, 21].

The aim of this study is to analyze prevalence and outcome of pregnancies conceived at extreme maternal ages (age equal or lower than 17 years old and equal or higher than 40 years old), and to verify the impact of maternal age on pregnancy outcomes.

## Methods

This retrospective study considered 22,933 single consecutive pregnancies carried out in our Obstetrics and Gynecology Clinic in University Hospital Santa Maria della Misericordia (tertiary referral center). The present study was approved by the internal review board, it was conducted in accordance with Helsinki Declaration and it followed the dictates of the general authorization to process personal data for scientific research purposes by the Italian Data Protection Authority. All women who delivered between January 2001 and December 2014 were eligible to be included in this study. Multiple pregnancies were excluded.

Maternal-fetal and neonatal data were gathered from clinical databases of our Obstetric and Neonatal facilities. Patients were categorized into 4 groups according to maternal age at delivery (< 17 years; 18–28 years; 29–39 years; > 40 years). Personal data assessed were: maternal age, parity, assisted reproduction techniques, provenance and education level (low schooling was considered below 8 years of study). Pregnancy and neonatal outcomes assessed were: gestational age at delivery, fetal presentation, mode of onset of labour, mode of delivery, pregnancy-related hypertensive disorders (PRHDs), gestational diabetes mellitus (GDM), gestational age at birth, neonatal length, neonatal cranial circumference, placental weight, neonatal sex, neonatal weight, apgar score at first and fifth minute, eventual administration of prenatal corticosteroid profilaxis, intrauterine growth restriction (IUGR), small for gestational age (SGA), large for gestational age (LGA), presence of neonatal congenital anomalies, neonatal distress syndrome (RDS), neonatal resuscitation (NR), neonatal intensive care unit (NICU) admission, periventricular leucomalacia (PVL), grade 3 and 4 intraventicular hemorrhage (IVH), retinopathy of prematurity (ROP), and perinatal death.

Gestational age was calculated by the date of the last known menstrual period and confirmed by ultrasound examination during first and second trimester. The presence of hypertension was defined as a systolic blood pressure ≥ 140 mmHg or a diastolic blood pressure ≥ 90 mmHg [22, 23]. The following items were grouped as PRHDs: eclampsia, pre-eclampsia, pre-eclampsia superimposed on chronic hypertension, and gestational hypertension [24, 25]. Pre-eclampsia was defined as hypertension accompanied by proteinuria first detected after 20 weeks gestation [22, 23]. Proteinuria was defined as the presence of urinary protein in concentrations more than 0.3 g in a 24-h period (this usually correlates with 30 mg/dl or greater in a random urine determination). Gestational hypertension was diagnosed in the same way as pre-eclampsia, but without proteinuria, and eclampsia was diagnosed in the same way as pre-eclampsia, but with seizures [22, 23]. Chronic hypertension was diagnosed when hypertension was present before the 20th week of pregnancy [22, 23]. IUGR was defined as the sonographic finding of foetal weight below the tenth percentile of expected weight for gestational age (using Hadlock formula), linked with the increased pulsatility index of umbilical artery greater than two standard deviations, and a post-partum verification with a foetal weight under the 10th centile at birth. In this study, SGA and LGA were defined as neonatal weight under the 10th and over the 90th percentiles for gestational age, respectively [26, 27]. Preterm delivery was defined as delivery occurring before 37 completed weeks of gestation. Over the course of our study period, the diabetes screening method was changed. Up until 2010 a universal 50 g oral glucose challenge was performed (as previously described [28]), between 2010 and 2011 the universal screening method changed as previously described [29, 30], and starting from 2011 gestational diabetes was diagnosed through the 75-g two-hour oral glucose tolerance test before or after 24 weeks of gestation in the second trimester according to the presence of risk factors [30, 31]. All foetuses in breech or abnormal presentation were delivered by cesarean section. A diagnosis of PVL was established on the basis of magnetic resonance imaging findings, routinely performed on the basis of Volpe’s study [32]. Grade 3 and 4 intraventicular hemorrhage (IVH) were diagnosed using cranial ultrasound and classified according to Papile’s definitions [33]. ROP was diagnosed by an experienced ophtamologist and classified according to the international classification of ROP [34]. Perinatal death was defined as death that occurs at less than 28 days of age and fetal deaths at a gestational age of 20 weeks or more [35].

Statistical analysis was performed using R software (version 3.4.1; R Core Team (2017). R: A language and environment for statistical computing. R Foundation for Statistical Computing, Vienna, Austria. URL https://www.R-project.org/.). *P* < 0.05 was considered statistically significant. Data are presented as median and interquartile range (IQR) for continuous non parametric variables; mean ± standard deviation for continuous parametric variables. Dicotomic variables were coded as percentage and absolute values, except for missing values (NA). The results of logistic regression models were presented as odds ratio (OR) at 95% confidence interval (CI). The distribution of continuous variables was evaluated with Kolmogorov-Smirnov test to detect whether the distribution was normal or not. For continuous variables the following statistical tests were employed: t-test for parametric variables and Wilcoxon test for continuous non parametric variables. Subsequently, logistic regression analysis was performed, considering established outcomes as dependent variables and the possible risk factors for such outcome as independent variables. In the multivariate model we took into account all possible predictive factor with *p* < 0.200 in univariate analysis. The Initial multivariate model included all variables and their interactions. When interactions turned out to be non significant, analysis without interaction model was used. In addition, the *p*-values of the multivariate analysis were adjusted using the false discovery rate test.

## Results

**Fig. 1:**
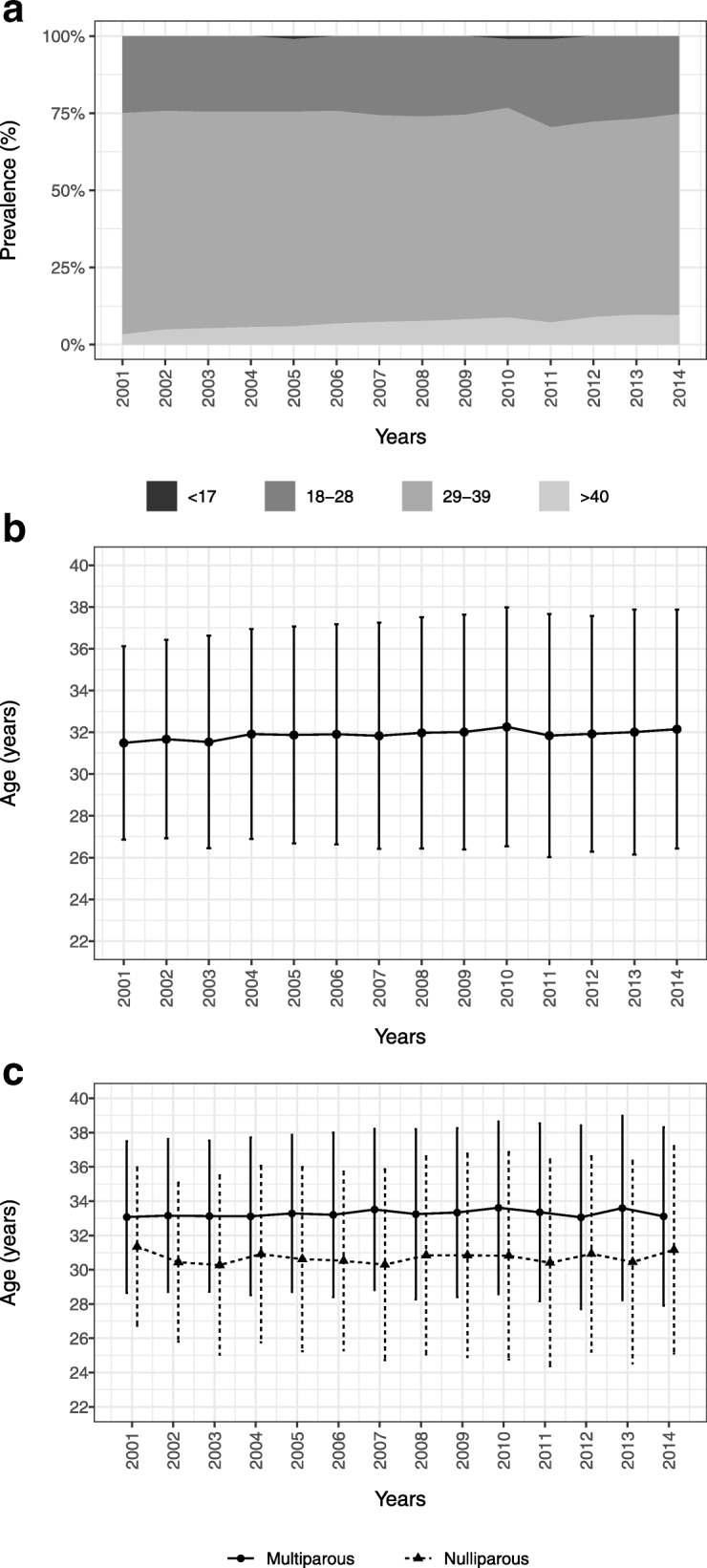
**a** Time trend of the age groups analyzed during the study period. **b** Time trend of Average age and relative standard deviation. **c** Time trend of Average age and relative standard deviation in nulliparous and pluriparous women

During the considered period 22,933 single pregnancies gave birth in our unit. The mean maternal age at delivery was 31.88 years (±5.38) and 55.88% of women were nulliparous. We recorded 71 women aged < 17 years, 5714 aged between 18 and 28 years, 15591 aged between 29 and 39 years, and 1552 aged > 40 years. Figure 1a shows the consistent increase of pregnant women over 40 years of age in our population from 2001, as well as the low prevalence of pregnancies in patients younger than 17 years of age. Figure 1b and c depict the yearly mean age at delivery, which was constantly rising, and the contribution of nulliparous and multiparous women (Fig. 1c). In particular there was a significant increase in maternal age by 0.041 years (95% CI 0.024–0.058) in each year of the study.

Table 1 reports the characteristics of the population. Two elements were particularly relevant: preterm deliveries and PRHDs occurred more frequently among older women. In addition, low schooling was more frequent among younger women. Tables 2 and Additional file 1: Table S1 describe labour and delivery (in both global population and nulliparous women). It is worth noting that as maternal age increased, spontaneous labour and delivery declined significantly. GDM and abnormal fetal presentations (including breech presentation) were also more common among older women. Fetal outcomes are presented in Table 2 and Additional file 2: Table S2. Women over 40 years demonstrated a higher prevalence of RDS, grade 3 or 4 inter-ventricular hemorrhage, periventricular leucomalacia, neonatal cardio-pulmunar resuscitation, neonatal death and IUGR. In mothers under 17 years we observed a lower neonatal weight MoM and a greater incidence of grade 3 or 4 IVH.

**Table 1:** Characteristics of the study population categorised by age group

	< 17 years (71)	18–28 years (5714)	29–39 years (15591)	> 40 years (1552)	*p*
Maternal age (years)	16.52 (±0.63)	24.86 (±2.70)	33.59 (±2.89)	41.34 (±1.60)	1,2,3,4,5,6
Nulliparous women	98.55% (68/69)	69.84% (3858/5524)	52.31% (7897/15097)	38.22% (571/1494)	1,2,3,4,5,6
Low schooling	76.06% (54/71)	34.06% (1946/5714)	18.27% (2848/15591)	20.30% (315/1552)	1,2,3,4,5,6
Conception mode					
Spontaneous	98.59% (70/71)	99.46% (5683/5714)	98.40% (15342/15591)	96.13% (1492/1552)	4,5,6
Ovulation induction/IUI	1.41% (1/71)	0.35% (20/5714)	0.59% (92/15591)	0.52% (8/1552)	4
IVF/ICSI	0.00% (0/71)	0.19% (11/5714)	1.01% (157/15591)	3.35% (52/1552)	4,5,6
Gestational age at delivery (weeks)	38.80 (±2.56)	38.69 (±2.64)	38.61 (±2.50)	38.17 (±2.68)	3,4,5,6
Delivery < 32 weeks	2.82% (2/71)	3.65% (208/5705)	3.02% (470/15547)	3.80% (59/1552)	4
Delivery < 34 weeks	2.82% (2/71)	4.93% (281/5705)	4.55% (707/15547)	5.86% (91/1552)	6
Delivery < 37 weeks	8.45% (6/71)	9.87% (563/5705)	9.90% (1539/15547)	13.92% (216/1552)	5,6
Macro-region of origin					
Europe/Italy	80.00% (56/70)	81.29% (4637/5704)	92.03% (14339/15581)	94.71% (1469/1551)	2,3,4,5,6
Sub-Saharian Africa	12.86% (9/70)	6.54% (373/5704)	3.61% (562/15581)	2.45% (38/1551)	1,2,3,4,5,6
Arabian countries	1.43% (1/70)	5.84% (333/5704)	1.93% (301/15581)	1.42% (22/1551)	4,5
Other origin	5.71% (4/70)	6.33% (361/5704)	2.43% (379/15581)	1.42% (22/1551)	3,4,5,6
PRHDs	0.00% (0/71)	2.22% (127/5714)	2.51% (391/15591)	5.41% (84/1552)	3,5,6
GDM	0.00% (0/71)	2.54% (145/5714)	4.28% (667/15591)	6.44% (100/1552)	3,4,5,6

*Acronyms*: *IUI* Intrauterine insemination, *IVF* In vitro fertilization, *ICSI* Intracytoplasmatic sperm injection, *PRHD* Pregnancy related hypertensive disorder, *GDM* Gestational diabetes mellitus. Different numbers indicate statistically significant differences (*p* < 0.05): (1) < 17 years vs 18–28 years; (2) < 17 years vs 29–39 years; (3) < 17 years vs > 40 years; (4) 18–28 years vs 29–39 years; (5) 18–28 years vs > 40 years; (6) 29–39 years vs > 40 years. NS= no significant difference

**Table 2:** Labor, delivery, and neonatal characteristics and neonatal outcomes

	< 17 years (71)	18–28 years (5714)	29–39 years (15591)	> 40 years (1552)	*p*
Labor and delivery					
Abnormal fetal presentation	4.23% (3/71)	4.34% (248/5713)	5.33% (831/15590)	6.12% (95/1552)	4,5
Mode of labor					
Spontaneous	71.21% (47/66)	68.10% (3399/4991)	63.22% (8478/13411)	52.95% (726/1371)	3,4,5,6
Induced/augmented	19.70% (13/66)	22.30% (1113/4991)	21.19% (2842/13411)	19.69% (270/1371)	5
Without labor	9.09% (6/66)	9.60% (479/4991)	15.59% (2091/13411)	27.35% (375/1371)	3,4,5,6
Childbirth mode					
Operative vaginal delivery	5.63% (4/71)	8.58% (490/5714)	7.25% (1130/15591)	5.73% (89/1552)	4,5,6
Spontaneous delivery	77.46% (55/71)	67.12% (3835/5714)	60.77% (9474/15591)	48.84% (758/1552)	2,3,4,5,6
Caesarean section	16.90% (12/71)	24.31% (1389/5714)	31.99% (4987/15591)	45.43% (705/1552)	2,3,4,5,6
Caesarean section in labour	8.33% (5/60)	14.30% (645/4512)	16.72% (1893/11320)	22.09% (220/996)	3,4,5,6
Neonatal charateristics and outcomes					
RDS prophylaxis					
Not performed	97.18% (69/71)	96.04% (5488/5714)	96.34% (15020/15591)	94.33% (1464/1552)	5,6
Uncompleted	2.82% (2/71)	0.88% (50/5714)	0.66% (103/15591)	0.84% (13/1552)	2
Completed	0.00% (0/71)	3.08% (176/5714)	3.00% (468/15591)	4.83% (75/1552)	5,6
Neonatal male sex	49.30% (35/71)	51.40% (2937/5714)	50.86% (7930/15591)	51.29% (796/1552)	NS
1st minute Apgar score	8.00 (8.00–9.00)	8.00 (8.00–9.00)	8.00 (8.00–9.00)	8.00 (8.00–9.00)	NS
5th minute Apgar score	9.00 (9.00–9.00)	9.00 (9.00–9.00)	9.00 (9.00–9.00)	9.00 (9.00–9.00)	NS
Neonatal congenital malformation	4.23% (3/71)	1.38% (79/5714)	1.50% (234/15591)	2.06% (32/1552)	1
RDS	7.04% (5/71)	5.71% (326/5714)	5.71% (890/15591)	7.80% (121/1552)	5,6
Retinopathy of prematurity	1.41% (1/71)	0.82% (47/5714)	0.90% (140/15591)	1.10% (17/1552)	NS
3rd 4th degree IVH	1.41% (1/71)	0.26% (15/5714)	0.19% (29/15591)	0.52% (8/1552)	2,6
Periventricular leucomalacia	0.00% (0/71)	0.16% (9/5714)	0.08% (12/15591)	0.32% (5/1552)	6
NR	5.63% (4/71)	3.45% (197/5714)	3.30% (515/15588)	4.38% (68/1552)	6
NICU hospitalization	0.00% (0/71)	0.02% (1/5711)	0.03% (5/15582)	0.00% (0/1551)	NS
Perinatal death	0.00% (0/71)	0.93% (53/5714)	0.74% (116/15591)	1.16% (18/1552)	NS
IUFD	0.00% (0/71)	0.30% (17/5714)	0.37% (58/15591)	0.45% (7/1552)	NS
Neonatal death	0.00% (0/71)	0.63% (36/5714)	0.37% (58/15591)	0.71% (11/1552)	4,6

*Acronyms*: *RDS* Respiratory distress syndrome, *IVH* Intraventricular hemorrhage, *NR* Neonatal resuscitation, *NICU* Neonatal intensive care unit, *IUFD* Intrauterine fetal demise. Different numbers indicate statistically significant differences (*p* < 0.05): (1) < 17 years vs 18–28 years; (2) < 17 years vs 29–39 years; (3) < 17 years vs > 40 years; (4) 18–28 years vs 29–39 years; (5) 18–28 years vs > 40 years; (6) 29–39 years vs > 40 years. NS= no significant difference

Maternal age was an independent risk factor for the considered variables in multivariate analysis, as explained in Tables 3 and 4. In conclusion, multivariate analysis showed that maternal age over 40 years was an independent risk factor for preterm delivery, PRHDs, GDM, abnormal fetal presentation, cesarean section, and fetal PVL. We also observed that maternal age under 17 years or over 40 years was an independent risk factor for grade 3 or 4 neonatal IVH. Also after adjustment of the multivariate analysis *p*-values with the false discovery rate test maternal age over 40 years remained a significant risk factor for many of the previously listed outcomes, only fetal PVL *p*-value shifted slightly above the significant threshold.

**Table 3:** Risk factors for the following specified pregnancy outcomes considered as dependent variables: delivery < 37 weeks’ gestation; pregnancy related hypertensive disorders; and caesarean section. Possible risk factors were considered as independent variables. Univariate and multivariate (*) logistic regression analysis were performed. (**) Excluded from the analysis non cephalic fetal presentation. (#) *p*-value corrected for the false discovery rate test

	OR (CI95%)	*p*	OR (CI95%) (*)	*p* (*)	*p*(#)
Delivery < 37 weeks’ gestation					
Maternal age > 40 years	1.47 (1.27–1.71)	< 0.05	1.36 (1.16–1.61)	< 0.05	< 0.05
Nulliparous women	1.26 (1.16–1.38)	< 0.05	1.31 (1.19–1.44)	< 0.05	< 0.05
Conception via assisted reproductive techniques	1.53 (1.13–2.07)	< 0.05	1.24 (0.9–1.72)	0.180	0.184
PRHDs (ŧ)	10.82 (9.16–12.78)	< 0.05	12.25 (10.02–14.99)	< 0.05	< 0.05
Low schooling (ŧ)	1.25 (1.14–1.38)	< 0.05	1.31 (1.17–1.46)	< 0.05	< 0.05
Macro-region of origin					
Europe/Italy	Reference		Reference		
Sub-Saharian Africa	1.43 (1.19–1.73)	< 0.05	1.19 (0.97–1.47)	0.090	0.106
Arabian countries	0.75 (0.56–1)	< 0.05	0.75 (0.55–1.01)	0.060	0.077
Other origin	1.38 (1.11–1.71)	< 0.05	1.35 (1.08–1.69)	< 0.05	< 0.05
Pregnancy related hypertensive disorders					
Maternal age > 40 years	2.32 (1.83–2.94)	< 0.05	2.36 (1.86–3.00)	< 0.05	< 0.05
Nulliparous women	1.07 (0.91–1.26)	0.439			
Conception via assisted reproductive techniques	2.4 (1.53–3.75)	< 0.05	2.28 (1.45–3.58)	< 0.05	< 0.05
Low schooling	1.53 (1.28–1.82)	< 0.05	1.4 (1.17–1.69)	< 0.05	< 0.05
Sub-Saharian Africa origin	2.73 (2.08–3.58)	< 0.05	2.53 (1.91–3.36)	< 0.05	< 0.05
GDM					
Maternal age > 40 years	1.74 (1.41–2.16)	< 0.05	1.71 (1.37–2.12)	< 0.05	< 0.05
Nulliparous women	0.71 (0.62–0.82)	< 0.05	0.74 (0.64–0.84)	< 0.05	< 0.05
Conception via assisted reproductive techniques	2.02 (1.35–3.03)	< 0.05	2.12 (1.4–3.19)	< 0.05	< 0.05
Low schooling	1.08 (0.93–1.27)	0.309			
Macro-region of origin					
Europe/Italy	Reference		Reference		
Sub-Saharian Africa	1.44 (1.08–1.91)	< 0.05	1.44 (1.08–1.93)	< 0.05	< 0.05
Arabian countries	1.5 (1.07–2.11)	< 0.05	1.49 (1.05–2.1)	< 0.05	< 0.05
Other origin	1.35 (0.97–1.87)	0.077	1.33 (0.94–1.86)	0.104	0.104
Abnormal fetal presentation					
Maternal age > 40 years	1.22 (0.98–1.52)	0.068	1.29 (1.03–1.61)	< 0.05	< 0.05
Nulliparous women	1.53 (1.35–1.73)	< 0.05	1.52 (1.34–1.73)	< 0.05	< 0.05
Conception via assisted reproductive techniques	1.94 (1.34–2.8)	< 0.05	1.65 (1.14–2.39)	< 0.05	< 0.05
Arabian countries origin	0.37 (0.21–0.64)	< 0.05	0.37 (0.21–0.66)	< 0.05	< 0.05
Caesarean section (**)					
Maternal age > 40 years	2.03 (1.82–2.27)	< 0.05	1.99 (1.78–2.23)	< 0.05	< 0.05
Nulliparous women	1.01 (0.95–1.07)	0.774			
Conception via assisted reproductive techniques	1.68 (1.34–2.12)	< 0.05	1.52 (1.20–1.93)	< 0.05	< 0.05
PRHDs (ŧŧ)	4.74 (3.97–5.65)	< 0.05	29.6 (10.72–81.70)	< 0.05	< 0.05
Low schooling	1.07 (1.00–1.15)	< 0.05	1.02 (0.95–1.10)	0.610	0.612
Sub-Saharian Africa origin	1.61 (1.45–1.79)	< 0.05	1.63 (1.46–1.82)	< 0.05	< 0.05
Induced/augmented labor	0.97 (0.89–1.05)	0.400			
Neonatal weight (MoM) (ŧŧ)	0.68 (0.55–0.85)	< 0.05	0.89 (0.71–1.12)	0.330	0.379

*Acronyms*: *PRHD* Pregnancy related hypertensive disorder, *MoM* Multiple of median. Interaction terms: (ŧ) Interaction term for PRHDs and Low schooling OR 0.56 (CI95% 0.39–0.81) (*p* < 0.05); (ŧŧ) Interaction term for PRHDs and neonatal weight MoM OR 0.13 (CI95% 0.05–0.37)

**Table 4:** Risk factors for the following specified neonatal outcomes considered as dependent variables: Fetal PVL and fetal IVH (3rd-4th degree). Possible risk factors were considered as independent variables. Univariate and multivariate (*) logistic regression analysis was performed. (#) *p*-value corrected for the false discovery rate test

	OR (CI95%)	*p*	OR (CI95%) (*)	*p* (*)	*p*(#)
Fetal PVL					
Maternal age > 40 years	3.29 (1.24–8.73)	< 0.05	3.32 (1.17–9.44)	< 0.05	0.056
Nulliparous women	0.92 (0.43–1.99)	0.835			
Conception via assisted reproductive techniques	2.65 (0.36–19.65)	0.339			
Low schooling	2.15 (0.98–4.75)	0.057	1.22 (0.52–2.88)	0.650	0.754
Sub-Saharian Africa/Arabian/Other origin	3.8 (1.65–8.75)	< 0.05	2.71 (1.07–6.82)	< 0.05	0.061
PRHDs	6.54 (2.25–19.03)	< 0.05	1.06 (0.35–3.26)	0.920	0.918
Gestational age at birth (weeks) (ŧ)	0.68 (0.63–0.72)	< 0.05	0.66 (0.6–0.73)	< 0.05	< 0.05
Prenatal RDS prophylaxis (ŧ)	7.42 (4.95–11.13)	< 0.05	0.06 (0.00–1.00)	0.050	0.070
Fetal IVH (3rd-4th degree)					
Maternal age < 17 years or > 40 years	2.69 (1.31–5.53)	< 0.05	2.97 (1.24–7.14)	< 0.05	< 0.05
Nulliparous women	1.49 (0.84–2.64)	0.17			
Conception via assisted reproductive techniques	2.61 (0.63–10.75)	0.185			
Low schooling	1.49 (0.83–2.68)	0.184			
Sub-Saharian Africa origin	2.86 (1.22–6.71)	< 0.05	1.59 (0.59–4.28)	0.360	0.481
PRHDs	2.99 (1.08–8.33)	< 0.05	0.71 (0.24–2.1)	0.530	0.533
Gestational age at birth (weeks)	0.62 (0.58–0.66)	< 0.05	0.62 (0.58–0.66)	< 0.05	< 0.05

*Acronyms*: *PRHD* Pregnancy related hypertensive disorder, *PVL* Periventricular leucomalacia, *RDS* Respiratory distress syndrome, *IVH* Intraventricular hemorrhage. Interaction terms: (ŧ) Interaction term for gestational age at birth and prenatal RDS prophylaxis OR 1.12 (CI95% 1.02–1.23) (*p* < 0.05)

## Discussion

Our study shows an increasing prevalence of mothers aged over 40 years. This is consistent with Italian and European demographic data, in which Italy ranks as one of the top countries for high maternal mean age at birth of the first child [1, 2]. In all developed countries, the deep social and economic transformations of recent decades have led to higher life standards together with a deferment of parenthood [3–5, 36]. This phenomenon is due to multiple determinants: global aging of population with an increasing prevalence of women aged between 35 and 45 years, the change in social customs with a rise in divorces and second marriages, improvements to women’s educational and professional outlooks as well as the diffusion of contraception. The availability of contraception has made women protagonists of their childbearing options. Asked to explain the main determinants of their pregnancy plan, a survey group of women mentioned educational and career achievements, financial goals and emotional stability, illustrating how individual readiness seems to be an essential factor in guiding childbearing options [37].

In our study the incidence of PRHDs is higher as maternal age increases. This is in line with the findings of other studies. In a retrospective analysis of 8′079’996 live births of singletons the adjusted odds ratio of pregnancy associated hypertension for women ≥45 years, compared with women aged 30–34, was 1.55 and 2.13 respectively for primiparas and multiparas [38]. Another case control study found a significantly higher rate of preeclampsia in women over 45 years compared to the control group (10.7% versus 1.8% respectively) [39].

The higher risk of developing GDM with advancing age that we confirmed is widely reinforced in literature, and can be explained by the progressive depletion of pancreatic β-cell function that leads to a reduced insulin sensitivity. This data justifies the universal screening in all pregnant women over 35 years old that we currently apply [15, 38, 39].

Maternal age over 40 years is an independent risk factor for preterm delivery. This data was reported in various population-based studies [40–42]. Swedish research studying 2′009’068 birth found that advanced maternal age is associated with an increased risk of preterm birth, especially very preterm birth, irrespective of parity, (adjusted ORs 1.18 to 1.28 at 30–34 years, from 1.59 to 1.70 at 35–39 years, and from 1.97 to 2.40 at ≥40 years) [43]. Another population based cohort study from Sweden restricted to 173,715 healthy women with a single pregnancy, reported increasing adjusted odds ratios for preterm delivery with increasing maternal age, after adjusting for demographic characteristics, smoking, history of infertility, and other medical conditions [44]. The underling reasons are not clear. One of the mechanisms that could be involved is placental vascular pathology. In fact, spontaneous preterm birth has been associated with a four- to seven-fold increased risk of histological evidence of placental bleeding, loss of vessel integrity and lack of physiologic conversion of maternal spiral arteries [45]. Preeclampsia and hypertensive disorders are also associated with uteroplacental ischemia and with pre-term birth. As we illustrated, older women have a higher risk of both these complications, and are also more likely to have a IUGR, which is also vascular-mediated. Another possible actor in the pathway of preterm birth in older women is progesterone deficiency: progesterone levels decrease with maternal age. Low levels of this hormone are associated with preterm delivery and progesterone supplementation has been proven to be effective in preventing it [46].

We demonstrated a lower prevalence of labour and spontaneous delivery in women of advanced age, and a higher rate of cesarean section. A lot of other studies came to similar conclusions [47–51]. In a retrospective cohort study of mothers aged 45 years or more the cesarean section rate was 49% compared to 23% in the 20–29 year age group (*p* < 0.001) [47]. In another retrospective analysis, among 77 women aged 50 years or older, who conceived after in vitro fertilization with donor oocytes, 68% were delivered by cesarean section [51]. Another paper that reviewed 24′032 pregnancies of women who delivered at age 40 or over, illustrated a higher risk of operative delivery (cesarean, forceps, and vacuum deliveries): 61% compared to the 35% in younger nulliparous women, in spite of lower birth weight and gestational age [50]. In a cohort study on 78′880 singleton births in the United States, which excluded patients with prior cesarean section, the proportions and the risks of primary cesarean section increased with age in spite of parity [49]. The reasons for the high rate of operative delivery in older women are controversial. In our sample a higher rate of cesarean section in older women remains even after controlling for labour induction and exluding abnormal fetal presentations, suggesting that beyond comorbilities other factors impact on this surgical intervention. Labour distocia has been reported to be significantly higher in advanced age [52]. The correlation between uterine dysfunction and age has already been studied, and seems to have a linear increase during childbearing years [53]. In particular our data confirms a significant association between abnormal fetal presentation and older age [54], which could also be due to impaired placentation correlated with advanced maternal age [45, 55].

A maternal age over 40 years was an independent risk factor for fetal PVL. An age either below 17 years or over 40 years was an independent risk factor for grade 3 or 4 neonatal IVH. These results describe a previously unknown important impact of maternal age on neonatal morbidity that, to our knowledge, we disclose for the first time. The pathogenesis of IVH is linked to the fragility of the germinal matrix in immature preterm infants, on which an alteration of cerebral blood flow related to hypoxia-ischemia and reperfusion intervenes [56]. Therefore, we hypothesize that the implied mechanism in women at the extremes of childbearing age has, again, a vascular origin. In fact, an impaired vascular adaptation has been considered also in adolescent pregnancies [57]. Physical immaturity in younger mothers would be an obstacle to the physiological placental invasion through multiple pathways: an incomplete estrogen-dependent growth of the uterus, a residual ontogenetic progesterone resistance and a deficient tissue specific programming of immune cells.

The main limitation of this study is its retrospective design. Meanwhile, its strengths lie firstly in the fact that it allows the possibility to take important confounding factors into account, and secondly that clinical management was handled by the same team following the same obstetrical and neonatal policies to manage all cases. In addition, in comparison to recent literature where the mean age observed was 30.65 years (95% CI 28.56–32.75) [11, 12, 17, 39, 43, 49], our study with a mean maternal age of 31.88 years (±5.38) better contributes to evidence-based knowledge of possible risk factors of delayed childbearing in a aging population.

## Conclusions

In spite of the underlying mechanisms, these findings confirm the negative impact of extreme maternal ages on pregnancy. As the trend of delaying childbearing is well-established and likely to continue, these results should be carefully taken into account by maternal care providers in order to inform women adequately, providing evidence-based knowledge to support their procreation choices, and to improve clinical surveillance aiming to identify early signs of adverse outcomes.

## Additional file

Additional file 1: **Table S1.** Labor and delivery characteristics in nulliparous women. (DOCX 14 kb)

Additional file 2: **Table S2.** Neonatal anthropometric measurements. (DOCX 14 kb)

## Data Availability

The data that support the findings of this study are available, but restrictions apply to the availability of these data, which was used under license for the current study, and so are not publicly available. Data are however available from the authors upon reasonable request and with permission of the Internal Review Board.

## Abbreviations

GDM: Gestational diabetes mellitus
ICSI: Intracytoplasmatic sperm injection
IUFD: Intrauterine fetal demise
IUGR: Intrauterine growth restriction
IUI: Intrauterine insemination
IVF: In vitro fertilization
IVH: Intraventricular hemorrhage
LGA: Large for gestational age
MoM: Multiple of median
NICU: Neonatal intensive care unit
NR: Neonatal resuscitation
PRHD: Pregnancy related hypertensive disorder
PVL: Periventricular leucomalacia
RDS: Respiratory distress syndrome
SGA: Small for gestational age
