# Supplementary material for: Maternal age and the risk of adverse pregnancy outcomes: a retrospective cohort study
Source: BMC Pregnancy Childbirth. 2019 Jul 23;19:261. doi: 10.1186/s12884-019-2400-x (PMC6651936; doi:10.1186/s12884-019-2400-x)
Supplement: Supplementary file 1 — Table S1. Labor and delivery characteristics in nulliparous women. (DOCX 14 kb) [file 12884_2019_2400_MOESM1_ESM.docx]

**Additional file 1: Table S1**- Labor and delivery characteristics in nulliparous women.

|  | <17 years (68) | 18-28 years (3858) | 29-39 years (7897) | >40 years (571) | p |
| --- | --- | --- | --- | --- | --- |
| Abnormal fetal presentation | 4.41% (3/68) | 4.49% (173/3857) | 6.72% (531/7896) | 7.71% (44/571) | 4,5 |
| Mode of labor |  |  |  |  |  |
| Spontaneous | 70.77% (46/65) | 66.27% (2234/3371) | 59.42% (3865/6504) | 45.47% (221/486) | 3,4,5,6 |
| Induced/augmented | 20.00% (13/65) | 26.37% (889/3371) | 28.95% (1883/6504) | 28.81% (140/486) | 4 |
| Without labor | 9.23% (6/65) | 7.36% (248/3371) | 11.62% (756/6504) | 25.72% (125/486) | 3,4,5,6 |
| Childbirth mode |  |  |  |  |  |
| Operative vaginal delivery | 5.88% (4/68) | 11.38% (439/3858) | 11.38% (899/7897) | 10.68% (61/571) | NS |
| Spontaneous delivery | 76.47% (52/68) | 63.76% (2460/3858) | 54.96% (4340/7897) | 37.48% (214/571) | 1,2,3,4,5,6 |
| Caesarean section | 17.65% (12/68) | 24.86% (959/3858) | 33.66% (2658/7897) | 51.84% (296/571) | 2,3,4,5,6 |

NOTES. Different numbers indicate statistically significant differences (p<0.05): (1) <17 years vs 18-28 years; (2) <17 years vs 29-39 years; (3) <17 years vs >40 years; (4) 18-28 years vs 29-39 years; (5) 18-28 years vs >40 years; (6) 29-39 years vs >40 years. NS=  no significant difference.
