# Supplementary material for: Maternal age and the risk of adverse pregnancy outcomes: a retrospective cohort study
Source: BMC Pregnancy Childbirth. 2019 Jul 23;19:261. doi: 10.1186/s12884-019-2400-x (PMC6651936; doi:10.1186/s12884-019-2400-x)
Supplement: Supplementary file 2 — Table S2. Neonatal anthropometric measurements. (DOCX 14 kb) [file 12884_2019_2400_MOESM2_ESM.docx]

**Additional file 2: Table S2**- Neonatal anthropometric measurements.

|  | <17 years (71) | 18-28 years (5714) | 29-39 years (15591) | >40 years (1552) | p |
| --- | --- | --- | --- | --- | --- |
| Neonatal weight (grams) | 3233.41 (±538.14) | 3251.12 (±640.17) | 3241.90 (±627.85) | 3163.41 (±678.23) | 5,6 |
| Neonatal weight (MoM) | 0.98 (±0.10) | 1.01 (±0.16) | 1.01 (±0.14) | 1.01 (±0.14) | 2,3 |
| Neonatal weight <1500 grams | 2.82% (2/71) | 3.22% (184/5714) | 2.88% (449/15591) | 3.67% (57/1552) | NS |
| Neonatal weight <2500 grams | 4.23% (3/71) | 8.16% (466/5714) | 8.49% (1323/15591) | 11.53% (179/1552) | 5,6 |
| SGA <3rd percentile | 1.41% (1/71) | 2.64% (151/5714) | 2.17% (338/15591) | 2.45% (38/1552) | 4 |
| SGA <10th percentile | 7.04% (5/71) | 9.36% (535/5714) | 8.34% (1301/15591) | 9.28% (144/1552) | 4 |
| LGA >90th percentile | 5.63% (4/71) | 11.83% (676/5714) | 12.24% (1908/15591) | 13.02% (202/1552) | NS |
| LGA >97th percentile | 1.41% (1/71) | 4.74% (271/5714) | 5.14% (802/15591) | 6.19% (96/1552) | 5 |
| IUGR | 1.41% (1/71) | 1.65% (94/5714) | 1.46% (227/15591) | 2.19% (34/1552) | 6 |
| Placental weight (grams) | 582.54 (±103.42) | 589.99 (±133.17) | 588.23 (±128.54) | 585.76 (±140.18) | NS |
| Neonatal length (cm) | 49.58 (±3.07) | 49.48 (±3.66) | 49.50 (±3.47) | 49.06 (±3.70) | 5,6 |
| Newborn head circumference (mm) | 339.90 (±20.53) | 340.55 (±24.15) | 341.09 (±22.27) | 339.48 (±23.95) | 6 |

NOTES. **Acronyms**: MoM = multiple of median; SGA = small for gestational age; LGA = large for gestational age; IUGR = intrauterine growth restriction. Different numbers indicate **statistically significant** differences (p<0.05): (1) <17 years vs 18-28 years; (2) <17 years vs 29-39 years; (3) <17 years vs >40 years; (4) 18-28 years vs 29-39 years; (5) 18-28 years vs >40 years; (6) 29-39 years vs >40 years. NS=  no significant difference.
